# Improving the Quality of Life of a Kidney Transplant Patient With Limb Edema: Amplatzer Vascular Plug Embolization of an Arteriovenous Fistula

**DOI:** 10.7759/cureus.63224

**Published:** 2024-06-26

**Authors:** Alexandra Esteves, Nuno A Oliveira, Emanuel Ferreira, Luís Rodrigues, Rui Alves

**Affiliations:** 1 Nephrology, Centro Hospitalar e Universitário de Coimbra, Coimbra, PRT

**Keywords:** limb edema, quality of life, kidney transplant, amplatzer® vascular plug ii, arteriovenous fistula

## Abstract

An arteriovenous fistula is the preferred vascular access option for hemodialysis patients. However, complications, such as high-output heart failure and upper limb edema due to central vein stenosis, may arise.

We describe a case of a 65-year-old kidney transplant patient with severe edema in the left arm due to central vein stenosis and ipsilateral umerocephalic arteriovenous fistula. He was a previous hemodialysis patient and received his kidney transplant in 2015.

This patient had an eight-month waiting list to undergo surgical ligation of the arteriovenous fistula. Since his quality of life was decaying, we decided to perform a peripheral vascular embolization with Amplatzer® vascular plugs (Abbott, Green Oaks, IL). After a two-month follow-up, the arm edema was significantly reduced, and no immediate complications were reported. This case highlights that the Amplatzer® vascular plug is a safe and effective alternative for arteriovenous fistula embolization in patients with arm edema due to central vein stenosis.

## Introduction

Hemodialysis is a lifesaving treatment that enables the prolongation of life in patients with end-stage kidney disease. According to the Kidney Disease Outcomes Quality Initiative (KDOQI) [1] and the “Fistula First” initiative, the preferred vascular access for hemodialysis is the arteriovenous fistula (AVF). In Portugal as of 2020, the prevalence of AVF was 73.4% [2] and the incidence was 38.2% [2]. However, the construction of an AVF creates an area of high flow that could lead to serious complications [3] such as high output heart failure (incidence unclear, between 9% and 24% [4,5]) and upper limb edema in the presence of central venous stenosis (described incidence between 4.3 and 13% [6,7]).

In patients with limb edema, quality of life can be harshly affected: there is decreased arm movement, diminished strength, and decreased ability to grip. In such patients, surgical occlusion of AVF is the preferred procedure. However, peripheral vascular embolization should be considered a viable option.

## Case presentation

We present a case of a 65-year-old man with severe edema in the left arm due to central vein stenosis and ipsilateral brachiocephalic AVF. This patient had a long-time history of chronic kidney disease (underwent hemodialysis for nine years) having done hemodialysis previously with a history of central venous catheters in both right and left internal jugular veins. He had been subjected to a kidney transplant in 2015. At 74 months post-transplant, he had a normal graft function with a serum creatinine of 0.8mg/dL at the time of referral. Since there was no foreseeable need for using the fistula for hemodialysis, and due to the high recurrence rate of stenosis of the central veins that would require endovascular balloon angioplasties, with a potential need for repeated procedures and exposure to both contrast and ionizing radiation, he was referred to Vascular Surgery and it was decided to ligate the AVF.

Facing an eight-month waiting list due to the COVID-19 pandemic, with increased limitations in his arm movements and decreased quality of life, we decided to perform a percutaneous plug occlusion using an Amplatzer® vascular plug (AVP) (Abbott, Green Oaks, IL). An AVP II [8] was chosen for the procedure. It is most commonly used for vascular conditions allowing for embolization of pulmonary arteriovenous malformations, aortoiliac aneurysms, internal AVFs (renal, carotid, subclavian, coronary, mesenteric), and varices (gastric, portal and mesenteric veins) [9]. This device consists of a nitinol self-expanding, multi-layered mesh, with six planes of cross-sectional coverage; this design is aimed at increasing density and flow disturbance within the lumen of the blood vessel, leading to the rapid formation of blood clots with subsequent rapid vascular occlusion. An 18 mm diameter (9-AVP2-018) was chosen after ultrasound evaluation of the cephalic vein internal diameter; it is important to choose an AVP with a diameter approximately 30%-50% larger than the vessel diameter at the occlusion site, to ensure proper anchoring of the plug and avoid migration.

After an ultrasound evaluation of the AVF, a retrograde puncture of the venous outflow was made with a 7F vascular sheath placement. An angiographic assessment was made, identifying the anastomosis of the fistula, between the cephalic vein and the brachial artery, and the tip of the vascular sheath was placed in the cephalic vein slightly distal to the anastomosis, at the intended deployment site of the AVP.

**Figure 1:**
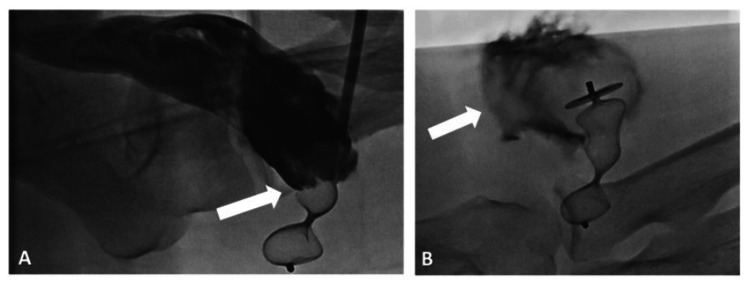
Fluoroscopy images of the Amplatzer ® Vascular Plug occluding the arteriovenous anastomosis of the AVF (A) and thrombi formation after the deployment of the plug (B)

The loading device of the AVP was inserted through the vascular sheath, and the AVP was then inserted at the proper location, at the extremity of the vascular sheath, using its delivery wire; the subsequent retraction of the vascular sheath allowed for the expansion and proper deployment of the AVP, in the cephalic vein, in a position immediately distal to the anastomosis; proper care was taken in order to avoid any extension into the brachial artery. With ultrasound and fluoroscopy (Figures 1A, 1B), we checked the correct positioning of the AVP to reduce the risk of complications: migration of the plug in case of insufficient diameter and persistent AVF flow due to early collateral veins.

Two minutes after the procedure was done, an ultrasound was used to confirm the disappearance of the AV flow and the maintenance of radial artery blood flow. We could also identify the formation of a thrombus in the cephalic vein of the left arm, thus confirming the juxta-artery occlusion of the cephalic vein.

**Figure 2:**
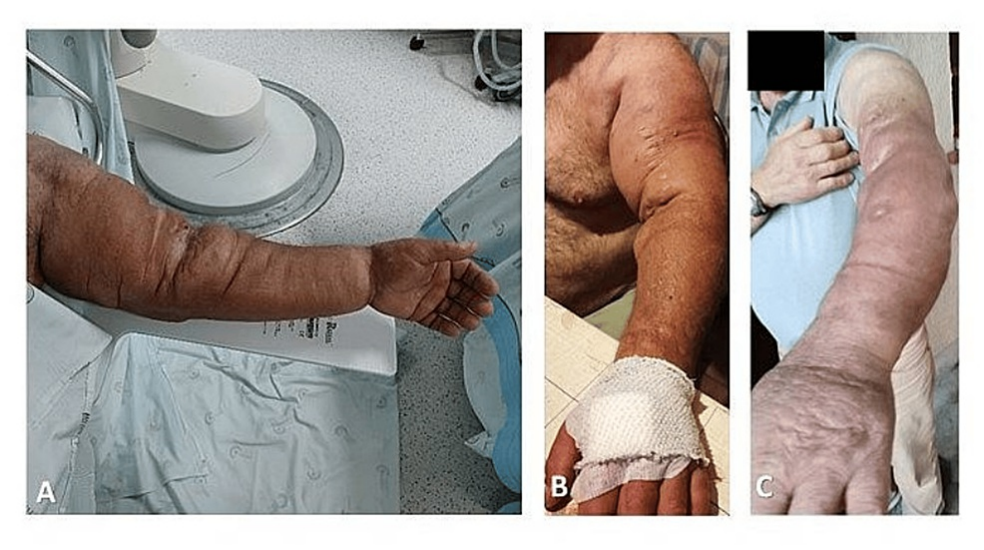
Patient’s left arm immediately before (A), immediately after (B) and two months after (C) the procedure

Two grams of cephazolin were administered and the patient was discharged with amoxicillin and clavulanic acid 875 mg +125 mg (twice a day for seven days). A significant reduction of arm edema was observed in the following weeks after the procedure. After two months, there were no complications reported (Figures 2A-2C), particularly no localized infections and there was no deterioration of renal graft function, with serum creatinine remaining within the normal range.

## Discussion

AVF can have many complications [3] (such as thrombosis, inflow and outflow stenosis, limb edema, and high output heart failure) which can diminish the patient's quality of life. Surgical occlusion is usually the standard treatment in patients when the AVF is no longer needed or presents clinically symptomatic dysfunction. However, in our hospital, particularly since the SARS-CoV-2 pandemic, it is a procedure with a moderate to long waiting list so that other treatment options may be explored.

To our knowledge, only a few articles in the literature reported using AVP for this purpose. An older case series published by Powell et al. [10] used AVP to occlude a high-flow AVF of seven patients, with a success rate of 100% and no complications after a follow-up period of three months. In 2017, Di Filippo et al. [11] reported a series of three cases in which AVP was used to occlude the AVF in patients with steal syndrome and arm edema: these had no complications (plug migration, ischemia, or access revascularization). Bourquelot et al. [12], published a study including 19 patients under hemodialysis in which AVP of different generations (I, II, IV) were used to occlude or reduce the flow of the AVF: the mean follow-up was 1.2 years, and no complications were observed.

Our case presents similar features to the reported literature [10-12]: the technique was similar, successful occlusion of the AVF was achieved and we also reported no complications during and after the procedure (at two months). However, Di Filippo et al. [11] reported that after the procedure one patient needed additional coiling to fully occlude the AVF, which was not necessary in our patient.

In most published case series, this procedure was made in patients undergoing hemodialysis or with end-stage kidney failure. Unlike the previous articles, our patient had a kidney transplant and was no longer on hemodialysis (he had a stable serum creatinine within the normal range). As far as we know, only Bourquelot et al. [12] used an AVP to occlude AVF in a kidney transplant patient, reporting a favorable outcome. However, the Amplatzer plug used was type I while we used type II, which has better occlusive properties due to the multi-layered mesh instead of the single mesh of the type I plug [8].

## Conclusions

In our case, the patient was an active man faced with progressively decreasing quality of life because of the increased arm edema. Since his waiting list period was over six months, we decided to place AVPs.

This patient’s edema reduced significantly, which led to an increased range of movements, better ability to grab objects, and reduced the probability of limb infections (the patient had previous cellulitis of this limb a year before this procedure) and, therefore, allowed for marked improvement in the patient quality of life. The use of the AVP allowed for a safe and faster resolution of this condition, with a minimally invasive procedure. Therefore, we believe this can be an effective alternative to open surgery when the need to ligate a dialysis AVF arises.
